# Representation of unplanned pregnancy in contemporary Spanish cinema: from voluntary abortion to unwanted motherhood

**DOI:** 10.1080/26410397.2026.2713898

**Published:** 2026-08-05

**Authors:** Mariona Visa Barbosa, Margarida Carnicé Mur, Joana Soto Merola

**Affiliations:** a Associate Professor, University of Lleida, Lleida, Spain. *Correspondence*: mariona.visa@udl.cat; b Lecturer, University of Lleida, Lleida, Spain

**Keywords:** contemporary Spanish cinema, abortion, motherhood, unplanned pregnancy

## Abstract

This article examines the representation of unplanned pregnancy in contemporary Spanish cinema between 2014 and 2024, with the aim of identifying the dominant narratives surrounding this experience. Drawing on a mixed-methods approach that combines quantitative and qualitative analysis of a selected corpus of films, the study investigates the narrative evolution of unplanned pregnancy, the diversity of the characters depicted, the reproductive agency of female protagonists, and the visibility of voluntary termination of pregnancy. The findings reveal that socio-economic conditions exert a decisive influence on how protagonists experience and negotiate unplanned pregnancy, shaping both their interpretive frameworks and their available choices. Despite this, the socio-demographic diversity of characters remains notably limited, particularly in relation to class, race, and other structural determinants, reflecting a homogenised representation of reproductive experiences in Spanish cinema. A key result of the study is the marginalisation of abortion as a reproductive option: legal abortion is explicitly represented in only two of the 14 films analysed, while most narratives emphasise either motherhood that is ultimately accepted or failed attempts to end the pregnancy. This pattern suggests that Spanish cinema continues to privilege conventional reproductive frameworks, offering few portrayals that normalise abortion as a legitimate and accessible choice. By critically engaging with these cinematic trends, the article contributes to broader debates on reproductive rights, gendered agency, and media representations of women’s health. It highlights the need for more diverse and realistic depictions of unplanned pregnancy to challenge dominant narratives and expand cultural understandings of reproductive justice.

## Plain language summary

This article analyses how contemporary Spanish cinema portrays unplanned pregnancy. We used a mixed-methods approach to examine how this situation is described in 14 feature films. We looked at how diverse the characters are and how visible abortion is. The characters’ economic situation had a strong effect on how they felt about unplanned pregnancy and dealt with it. The diversity of characters was very limited, especially in relation to class, race and economic status. Abortion was only included as an option in two of the films. Most of them showed the characters accepting motherhood, or failing to end the pregnancy. This suggests that Spanish cinema still favours conventional stories about reproduction. There was little mention of abortion as a legitimate and accessible choice. This article contributes to broader debates on reproductive rights, gender, and women's health in the media. It highlights the need for more diverse and realistic depictions of unplanned pregnancy. This would challenge dominant narratives and expand cultural understandings of reproductive justice.

## Résumé

Cet article examine la représentation des grossesses non désirées dans le cinéma espagnol contemporain entre 2014 et 2024, afin d’identifier les discours dominants entourant cette expérience. S’appuyant sur une approche à méthodes mixtes combinant analyse quantitative et qualitative d’un ensemble de films sélectionnés, l’étude explore l’évolution narrative des grossesses non désirées, la diversité des personnages représentés, le pouvoir d’agir des protagonistes féminines en matière de procréation et la visibilité de l’interruption volontaire de grossesse. Les résultats révèlent que les conditions socio-économiques exercent une influence déterminante sur la manière dont les protagonistes vivent et gèrent une grossesse non désirée, façonnant à la fois leurs cadres d’interprétation et les choix qui s’offrent à elles. Malgré cela, la diversité socio-démographique des personnages reste remarquablement limitée, notamment en ce qui concerne la classe sociale, la race et d’autres déterminants structurels, ce qui traduit une représentation homogénéisée des expériences reproductives dans le cinéma espagnol. Un résultat clé de l’étude est la marginalisation de l’avortement comme option reproductive: l’avortement légal n’est explicitement représenté que dans 11,8% des films analysés, alors que la plupart des récits mettent l’accent soit sur une maternité finalement acceptée, soit sur des tentatives infructueuses d’interrompre la grossesse. Ce constat suggère que le cinéma espagnol continue de privilégier les conceptions conventionnelles de la procréation, offrant peu de représentations qui normalisent l’avortement comme un choix légitime et accessible. En analysant de manière critique ces tendances cinématographiques, l’article contribue aux débats plus larges sur les droits reproductifs, l’autonomie genrée et les représentations de la santé des femmes dans les médias. Il souligne la nécessité de descriptions plus diverses et réalistes des grossesses non désirées afin de remettre en question les discours dominants et d’enrichir la compréhension culturelle de la justice reproductive.

## Resumen

Este artículo examina la representación del embarazo no planificado en el cine español contemporáneo entre 2014 y 2024, con el objetivo de identificar las narrativas dominantes en torno a esta experiencia. El estudio aplica un enfoque de métodos mixtos que combina análisis cuantitativo y cualitativo de un corpus seleccionado de películas para investigar la evolución narrativa del embarazo no planificado, la diversidad de los personajes representados, la agencia reproductiva de las mujeres protagonistas y la visibilidad de la interrupción voluntaria del embarazo. Los hallazgos revelan que las condiciones socioeconómicas ejercen una influencia decisiva sobre la manera en que las protagonistas experimentan y negocian el embarazo no planificado, lo que define tanto sus marcos interpretativos como sus opciones disponibles. No obstante, la diversidad sociodemográfica de los personajes continúa siendo notablemente limitada, en particular en relación con clase, raza y otros determinantes estructurales, lo que refleja una representación homogenizada de experiencias reproductivas en el cine español. Un resultado importante del estudio es la marginación del aborto como opción reproductiva: el aborto legal se representa explícitamente en solo el 11.8% de las películas analizadas, mientras que la mayoría de las narrativas hacen hincapié en la maternidad que finalmente es aceptada o en intentos fallidos por interrumpir el embarazo. Este patrón indica que el cine español continúa privilegiando los marcos reproductivos convencionales, ofreciendo pocas representaciones que normalizan el aborto como una opción legítima y accesible. Al analizar críticamente estas tendencias cinemáticas, el artículo contribuye a debates más amplios sobre los derechos reproductivos, la agencia condicionada por el género y las representaciones mediáticas de la salud de las mujeres. Destaca la necesidad de tener representaciones más diversas y realistas del embarazo no planificado para cuestionar las narrativas dominantes y ampliar la comprensión cultural de la justicia reproductiva.

## Introduction

Unplanned pregnancy is not only a private or individual experience, but also one shaped by broader social, cultural, legal, and health systems. Within sexual and reproductive health and rights (SRHR), access to abortion, reproductive autonomy, and maternal wellbeing are closely linked to issues of inequality, stigma, and justice.^1,2^ While these dimensions are often examined through legal or clinical frameworks, they are also mediated by cultural representation.

Cinema constitutes a particularly relevant medium for examining sexual and reproductive health norms, as audiovisual narratives play a central role in the production and circulation of social meanings. Media and communication studies have long emphasised that representation is not merely reflective, but actively contributes to shaping norms, identities, and expectations.^3,4^ Through storytelling, visual embodiment, and affective engagement, film enables audiences to encounter experiences that are often socially silenced or stigmatised – such as abortion or unplanned pregnancy – and to interpret them within specific moral and cultural frameworks. From a global health perspective, analysing these representations helps illuminate the symbolic dimensions of reproductive health – that is, the meanings, values, norms, and emotions culturally attached to reproductive experiences – which complement the biomedical and rights-based approaches promoted by international sexual and reproductive health and rights frameworks, particularly those developed by the World Health Organization.^5^ For example, while these frameworks recognise the right to make informed reproductive decisions free from coercion or discrimination, films may depict unplanned pregnancy as either a legitimate context for autonomous decision-making or as a moral failure requiring redemption, thereby shaping what reproductive trajectories become socially imaginable, normalised, or marginalised.^6,7^ In this sense, cinema operates as a key site where reproductive experiences are negotiated and offers a complementary lens for understanding how they are framed not only within policy and healthcare systems, but also through the cultural imaginaries that shape public understanding.^8,9^ Adopting a rights-based perspective, this study approaches reproductive decision-making not simply as a matter of individual choice, but as a process shaped by structural conditions that enable or constrain the exercise of sexual and reproductive rights.

Although this study focuses on Spanish cinema, its relevance extends beyond the national context. In recent years, Spanish audiovisual production has gained increasing international visibility through film festivals, global distribution networks, and streaming platforms, facilitating the transnational circulation of narratives and imaginaries.^10,11^ As a result, representations produced within the Spanish context participate in broader global media flows, contributing to cultural conversations about gender, reproductive rights, and social change across different contexts.^12^ Analysing Spanish cinema therefore offers insight not only into local dynamics, but also into how reproductive experiences are framed within wider transnational debates on sexual and reproductive health and rights.

In the last decade, numerous forms of cultural production have reflected a notable increase in stories centred on the experience of motherhood.^13,^^14^ In the case of Spanish audiovisual production, recent research^15^ has identified a growing presence of content addressing the reproductive process on streaming platforms. Based on a quantitative mapping and classification of titles released between 2014 and 2024, this study also pointed to a notable increase in narratives specifically dealing with abortion.

These developments have taken place alongside significant changes. In 2010, Spain adopted Law 2/2010, which established abortion on request during the first 14 weeks of pregnancy and regulated terminations up to 22 weeks under specific circumstances. The law also allowed minors aged 16 and 17 to access abortion without parental consent. In 2023, further reforms removed the mandatory three-day reflection period and restored minors’ right to decide without mandatory parental involvement.^16^ That same year, Spain recorded 102,102 abortions, corresponding to a rate of nearly 12 per 1000 women aged 15–44; 90. One percent were elective abortions and 70.3% were medication abortions.^17^

Within this context, marked by debates over reproductive rights, new family models, and an increasingly clear decline in the birth rates of Western countries, the representation of unplanned pregnancy in cinema takes on special relevance. Our research question focuses on how contemporary Spanish films about unplanned pregnancy (2014–2024) represent experiences of reproductive decision-making, and to what extent do these representations challenge or reinforce dominant social imaginaries. Our research had three objectives: to identify, catalogue, and describe the corpus of contemporary Spanish films (2014–2024) that depict unplanned pregnancy, including their main thematic and production characteristics; to examine the socio-demographic representation of protagonists and analyse the motivations shaping their decisions to continue or terminate the pregnancy, as well as their physical and emotional trajectories; and to analyse the narrative frameworks through which unplanned pregnancy is constructed, focusing on plot development and the meanings associated with different reproductive outcomes (e.g. motherhood, abortion, or frustrated termination).

The initial hypotheses are:

- The number of narratives about unplanned pregnancy has increased significantly in recent years, as has the socio-demographic diversity of their protagonists in terms of nationality, social class, and sexual orientation.
- There is a perceived increase in the decision-making power and agency of women protagonists regarding the evolution of their pregnancy.
- The depiction of unplanned pregnancies ending in voluntary termination of the pregnancy is more frequent, with a lower incidence of representations focused on negative consequences and a greater alignment with current health protocols.

### Theoretical framework

#### The question of wanting: a terminological debate

The term *unplanned pregnancy* is widely used in sexual and reproductive health (SRH), yet its meaning remains contested. Research has consistently shown that *unplanned*, *unintended* and *unwanted* pregnancy are not equivalent categories and are frequently used interchangeably despite referring to different dimensions of reproductive experience. While *unintended pregnancy* generally encompasses both mistimed and unwanted pregnancies, *unplanned pregnancy* refers more specifically to the absence of prior planning and does not necessarily reflect women's desires, emotions or subsequent evaluations of the pregnancy. Likewise, *unwanted pregnancy* should not be assumed to indicate a stable rejection of pregnancy, as reproductive intentions and feelings often change over time and are characterised by ambivalence, fluidity and contextual negotiation rather than fixed preferences.^18–23^

These conceptual distinctions have important analytical implications. Conventional classificatory frameworks, largely developed within biomedical and public health paradigms, prioritise standardised and measurable categories that may fail to capture the complexity of lived reproductive experiences. Rather than representing purely individual intentions, reproductive decisions are shaped by changing personal circumstances, social relationships and structural conditions, including economic precarity and unequal access to reproductive healthcare. This ambiguity is also evident in the Spanish context, where *unplanned* and *unwanted* pregnancy are frequently used interchangeably despite referring to different reproductive realities. Following recent scholarship, this article therefore uses the term *unplanned pregnancy* while recognising that reproductive trajectories cannot be reduced to fixed or homogeneous categories of intention or desire.^24–29^

This terminological choice reflects broader conceptual concerns. As discussed above, frameworks based on pregnancy intention often presuppose the desires of the pregnant person, whose subjectivity is frequently marginalised in dominant representations. As several authors have argued, the prevailing paradigm of pregnancy intention relies on assumptions of stable and measurable preferences, failing to capture the complexity, ambivalence, and contextual nature of reproductive experiences.^19,22,30^

#### The representation of unplanned pregnancy in cinema

Recent decades have seen a rise in studies on the representation of motherhood in cultural works,^31–46^ as well as on the representation of reproductive processes.^14,47,48^ Several studies have also explored the portrayal of abortion^7,49–53^ and the motivations behind its inclusion in plots.^8^

Gretchen Sisson and Katrina Kimport^7^ identified 169 films released between 1913 and 2012 that depicted unplanned pregnancies, noting significant changes post-1973, when Roe v. Wade legalised abortion in the United States (a decision recently overturned, shifting regulation back to individual states). Over the years, the number of unplanned pregnancies ending in voluntary termination has increased, with a 105% rise in films addressing this between 2003 and 2012 compared to the previous decade. For the most part, these were films in which abortion is part of a secondary plot (*The Godfather II*, 1974; *Dirty Dancing, 1987; 21 Grams*, 2003) and minor works, in which it is shown from the woman’s point of view.

The advent of over-the-counter pregnancy tests in the late twentieth century facilitated early pregnancy detection, influencing new pregnancy narratives in popular culture.^54^ Since the late 2000s, stories about unplanned pregnancies have expanded beyond romcoms to include genres like road movies, which focus on the journey to access legal abortion care, presenting pro-choice narratives where abortion is not portrayed as a traumatic experience. In Europe, female-directed films such as *Happening* (Diwan, 2021) and *Ninjababy* (Sve Flikke, 2021) have contributed to reframing reproductive health narratives in more realistic and diverse ways. This shift can also be observed in internationally produced films such as *Call Jane* (Nagy, 2022) and *Never, Rarely, Sometimes, Always.* (Hittman, 2020)

Abortion was first criminalised in Spain in 1822, briefly legalised by the Second Republic in 1937, and then banned again under Franco, forcing women to seek backstreet abortions or go abroad.^16^ The influence of National Catholicism continued to stigmatise abortion, which was partially decriminalised in 1985. In the 1960s, feminist debates led to films addressing abortion and imposed motherhood. The short film *Carmen de Carabanchel* (1965) depicted women’s despair over unwanted pregnancies, hinting at backstreet abortions. Francoist films, like *Aborto criminal* (1973), depicted abortion as a tragedy to restore moral order, while comedies like *Soltera y madre en la vida* (1969) stigmatised abortion as both a crime and sin.

In 1985, ten years after Franco’s death, Spain partially decriminalised abortion in cases of rape, fetal malformation, or danger to the mother's health. Quinqui cinema – a genre that emerged during Spain's democratic Transition, portraying the lives of marginalised working-class youth, delinquency, and social exclusion, often using non-professional actors – featured abortion as part of this broader social reality. Films such as *Colegas* (1982) showed abortion's impact on working-class youths. Art-house films, including Pedro Almodóvar’s *Entre tinieblas* (1983), used abortion to critique society. In the 1990s, despite a lack of legislative progress, social debates on reproductive rights expanded. In Solas,^55^ abortion is shown as a possible option for an unplanned pregnancy, although the character ultimately embraces motherhood.

While previous research has provided valuable insights into the representation of abortion and unplanned pregnancy, several gaps remain. Existing studies have often focused on Anglo-American contexts, with less attention to contemporary Spanish cinema as a distinct corpus. Moreover, abortion and motherhood are frequently analysed separately, rather than as part of a broader narrative process of unplanned pregnancy. Finally, there is limited research combining quantitative mapping with in-depth qualitative analysis to examine both the prevalence and narrative construction of these representations. In this context, the present study contributes to the field by offering a mixed-methods analysis of unplanned pregnancy in contemporary Spanish cinema. Focusing on films released between 2014 and 2024 enables us to examine these representations within a decade characterised by important legal, social, and audiovisual transformations in Spain.

## Methodology

A mixed-methods approach combining quantitative and qualitative techniques was used to achieve the proposed objectives. First, films were catalogued and the protagonists’ socio-demographic characteristics were analysed using quantitative content analysis. Then, a close-reading technique was applied to examine the narratives from an intersectional perspective.

The time frame (2014–2024) was selected to capture contemporary Spanish cinema. This period also coincides with renewed public and political debates on sexual and reproductive rights in Spain,^17,56^ within a broader global context of ongoing discussions around access to safe abortion and reproductive autonomy,^5^ making it particularly relevant for examining how reproductive decision-making is framed in recent audiovisual narratives.

The selected films were identified through a multi-step search strategy conducted between March and July 2024. The initial corpus was drawn from the MaterScreen Database,* a research tool developed within the MaterScreen project that catalogues around 1000 audiovisual works related to reproductive processes (e.g. menstruation, pregnancy, childbirth, abortion, postpartum, infertility, assisted reproduction, and menopause) across major video-on-demand platforms and Spanish broadcasters.

This database was complemented by targeted searches on streaming platforms (Movistar+, Netflix, Prime Video, Filmin, HBO Max, and Disney+) and on-demand services from Spanish broadcasters (RTVE Play, Atresplayer, Mitele, and 3Cat), using keywords such as *embarazo no planificado* (unplanned pregnancy) and *aborto* (abortion). In addition, supplementary searches were conducted through online sources (including film databases such as IMDb and FilmAffinity, as well as press articles and curated collections) to identify works that might not be indexed through platform search engines. When no direct results were found, related categories or curated lists (e.g. Filmin’s *Retratos alternativos de la maternidad*) were explored.

After an initial identification phase, a broader set of works was screened and coded. The final sample of 14 feature films was selected based on the following inclusion criteria: (1) Spanish production or co-production (with at least one Spanish production company involved); (2) release between 2014 and 2024; and (3) the presence of unplanned pregnancy or abortion as a central or significant narrative element. Works in which these themes appeared only marginally were excluded.

Identifying relevant works was challenging due to the absence of specific cataloguing systems for pregnancy-related films. Unlike other types of film classification, there is no distinct category or tag for pregnancy-related narratives,^14^ and keyword-based searches may overlook films in which reproductive processes are not explicitly indexed despite their narrative relevance.

A table was created to code the technical data and socio-demographic profiles of the protagonists based on previous analytical models,^7,53^ including technical information, socio-demographic characteristics, and the evolution of the unplanned pregnancy in the film. After coding, a final sample of 14 feature films was identified (Table 1).

**Table 1.** Technical details of the analysed films

Title	Director	Year	Synopsis
*La señora Brackets*... (Miss Brackets...)^a^	Sergio Candel	2014	Julia gives birth to Iván, but the father’s identity is unknown. Her mother rejects the child, while Julia's friend Marta offers support despite her own struggles. The babysitter, Carolina, also becomes affected by the dysfunctional dynamics.
*Hermosa juventud* (Beautiful Youth)	Jaime Rosales	2014	Two young lovers in their twenties struggle to make ends meet in crisis-ridden Spain. With limited resources and no major ambitions, they decide to make an amateur porn film to earn some money.
*La noche de las dos lunas* (The Night of the Two Moons)	Miguel Ferrari	2018	Three months pregnant, Federica learns her baby isn’t biologically hers due to an IVF mix-up. Although the woman who received her embryo had a miscarriage, Federica chooses to keep the baby. The biological parents then fight to reclaim it.
*Els dies que vindran* (The Days to Come)	Carles Marqués Marcet	2019	Vir and Lluís are a young couple from Barcelona who have only been dating for a year when they find out that ‘they’re pregnant’. For the next nine months, we follow them on their adventure, including the huge turn their lives will take, as they try to learn to be three before they’ve even had time to learn to be two.
*La inocencia* (Innocence)	Lucía Alemany	2019	Lis is a teenager who dreams of becoming a circus performer and leaving her hometown. She spends her days hanging out with her friends on the streets of her village and fooling around with her boyfriend. The lack of privacy and the townspeople’s constant gossip force Lis to keep their relationship secret.
*La hija* (The Daughter)	Manuel Martín Cuenca	2021	Fifteen-year-old Irene lives at a centre for juvenile delinquents. Newly pregnant, she is determined to turn her life around thanks to the encouragement of Javier, an educator at the centre. He invites her to stay with him and his wife Adela at their isolated home in the mountains while she carries her pregnancy to term. All he asks in return is that she agree to give them the baby.
*Madres paralelas* (Parallel Mothers)	Pedro Almodóvar	2021	Two single women who have both become pregnant by accident share a room at the hospital where they will give birth. While Janis is thrilled and has no regrets, teenaged Ana is scared and traumatised. Janis tries to cheer her up as they walk the hospital corridors. Their brief conversations will forge a bond between them that will last a lifetime.
*Paula*	Eugenio Canevari	2022	Paula works as a nanny for a wealthy soya-farming family deep in the province of Buenos Aires. When she discovers she is pregnant, she will have to silently put together a desperate solution.
*La jefa* (Under Her Control)	Fran Torres	2022	Sofía, a young employee at a multinational, unexpectedly gets pregnant. With no family in Spain and misgivings about motherhood, she seems doomed to give up her career and return to her country. Then her boss offers her a deal: let her adopt the child, and she will help Sofía advance her career.
*Culpa* (Guilt)	Ibon Cormenzana	2022	A woman is sexually assaulted by an acquaintance. Unable to cope with the situation, she leaves her whole life behind and retreats to a remote cabin in the mountains, cutting off all communication with her friends and loved ones, whilst an unwanted child grows inside her.
*La maternal* (Motherhood)	Pilar Palomero	2022	Fourteen-year-old Carla is defiant and rebellious. She lives with her mother in an old roadside restaurant, skipping school to spend time with a friend. When a social worker discovers that she is five months pregnant, Carla is admitted to a centre for underage mothers.
*Las buenas compañías* (In the Company of Women)	Sílvia Munt	2023	Summer of 1976. Sixteen-year-old Bea is working with a group of women to promote feminist causes and secure the legal right to abortion. Her political commitment and relationship with Miren will make this summer a key point in her life.
*O corno* (The Rye Horn)	Jaione Camborda	2023	Illa de Arousa, 1971. María harvests shellfish for a living. She also helps other women during childbirth. When a young woman dies during an abortion, she flees and embarks on a perilous journey in search of her freedom.
Mamífera (Female Mammal)	Liliana Torres	2024	Lola is enjoying life with her partner until an unexpected pregnancy turns her plans upside down. Although she has never wanted to be a mother, she faces social pressure and doubts in the run-up to her appointment at the clinic.

^a^ *La señora Brackets, la niñera, el nieto bastardo y Emma Suárez* (Miss Brackets, the Baby-sitter, the Bastard Grandson and Emma Suarez).

Next, a qualitative textual analysis was performed using the close-reading technique (Table 2). Close reading is widely applied in cultural studies in the context of contemporary popular culture^57,58^ and, in particular, in the analysis of fiction series and films.^59–61^ Close reading enables a detailed, in-depth examination of text and images, thereby facilitating the identification of patterns, themes, recurring motifs^62^ and underlying meanings in cultural content, whilst also helping to contextualise content within its cultural, historical and social setting. Following the analytical framework proposed by Buonanno^63^ and Fiske and Hartley,^64^ the analysis was structured around five complementary dimensions of audiovisual texts – context, mise-en-scène, narrative, content, and meanings (Table 2). For the purposes of this study, this framework was applied to analyse each film.

**Table 2.** Areas of analysis of the close reading

Area of analysis	Elements considered
Context	Socio-historical setting, location, environments and spaces. This also refers to the context of the production and of the work’s authors.
Story	Plot, characters, actions and temporal structure. Particular attention is paid to the story’s most important narrative threads. The main characters are identified and their actions, goals and plot resolutions are observed.
Content and Meanings	Themes, issues raised, subjects and institutions. The story’s sociological context and the values reaffirmed, rejected or transgressed in the work are considered. The main lines of conflict in the text are identified. This is related to the ideological code that gives the text coherence and situates it in a specific discourse.
Filmmaking	Formal, visual and sound elements. The technical code expressed through the construction of the shots, the pacing of the story achieved through editing, the lighting, the soundtrack, recurring visual motifs, etc.

### Reflexivity

This article is co-authored by three researchers affiliated with the University of Lleida (Spain). Mariona Visa and Marga Carnicé, both women and senior academics, are the principal investigators of the MaterScreen research project, focused on the analysis of the representation of reproductive processes (such as pregnancy, childbirth, abortion, and postpartum) in contemporary Spanish audiovisual fiction. Joana Soto, a researcher in an earlier stage of her academic career, contributed to the development of the database and the analysis. We acknowledge that our engagement with gender studies has led us to prioritise questions related to reproductive agency, the visibility of abortion, and the socio-cultural framing of motherhood. This perspective has also informed our attention to the structural conditions – such as class and access to resources – that shape reproductive decision-making in the films analysed. At the same time, our interpretations are informed by a critical awareness of the historical marginalisation and stigmatisation of abortion in Spanish and broader Southern European contexts. Finally, differences in seniority within the research team have contributed to combining consolidated theoretical frameworks with more exploratory analytical approaches.

## Results

### Production context of the films

All the films analysed are Spanish productions, except *Paula* (Eugenio Canevari 2022), a Spanish-Argentine co-production, and *La noche de las dos lunas* (Miguel Ferrari 2018), a Spanish-Venezuelan co-production. There has been an overall increase in films addressing unplanned pregnancy over time, although production has been irregular, with some years showing no films at all. A more noticeable rise can be observed from 2020 onwards, peaking in the 2022–2024 period. None of the film titles explicitly mention “pregnancy,” though some titles are related to motherhood (*La maternal, La hija, Madres paralelas, Mamífera*). Other titles reflect the protagonists’ emotions or themes like youth (*Hermosa juventud*), sisterhood (*Las buenas compañías*), and duality (*La noche de las dos lunas*). Notably, *O corno* references a historical method for inducing abortions, and *La señora Brackets, la niñera, el nieto bastardo y Emma Suárez* highlights family power dynamics. Of the 14 films, nine were directed by men and five by women, with women more frequently working as screenwriters. Filmin stands out as the platform hosting eight of the films. Most films are dramas (11 out of 14), with *Culpa* and *La hija* as thrillers, and *La señora Brackets * as a satirical comedy addressing family and social taboos about motherhood. The films are mostly set in contemporary Spain, except *O corno* and *Las buenas compañías*, set in the 1970s. The two co-productions are set in Argentina (*Paula*) and Venezuela (*La noche de las dos lunas*). In terms of protagonists’ age, the analysis considers a total of 14 female protagonists (one per film). Of these, six are minors, three are aged between 18 and 30, and five are over 30. All the women are white and heterosexual, with varied relationship statuses: six are in stable relationships, six are single, and some are unspecified. Professional stability is limited, with only two women in stable jobs, five unemployed, and three precariously employed.

### Unplanned pregnancy narratives

The analysed films resolve the story of the unplanned pregnancy through the following narrative possibilities:

(1) Wanted motherhood, arising from unplanned but ultimately accepted pregnancies (*Els dies que vindran, Madres paralelas, O corno, La noche de las dos lunas*). Despite being unexpected, the pregnancy is ultimately embraced. The protagonists are adult women, with some degree of emotional or financial stability, who manage to integrate motherhood into their life plans. In many cases, the initial conflict is transformed into reconciliation with the desire to mother. Although *La noche de las dos lunas* depicts a pregnancy that is initially planned, its inclusion responds to the way the film explores a mismatch between reproductive intention and outcome, prompting a process of renegotiation of motherhood and reproductive decision-making.
(2) Frustrated attempts to get an abortion, due to total or partial bans (*La maternal, Paula, Culpa*). In these films, there is an explicit desire to have an abortion, but the law or the healthcare context makes it impossible to do, either because abortion is criminalised or because the legal limit (14 weeks) has passed. The protagonists are usually teens with limited resources, a low level of education and no support networks. Their sociodemographic profile places them in a position of vulnerability conducive to the imposition of motherhood as the only possible way out.
(3) Legally and safely performed abortions (*Mamífera, La inocencia*). These narratives present abortion as a safe, legal and accessible option. In *Mamífera*, the process is shown as a straightforward medical procedure undergone by a highly-educated, middle-class, urban woman. However, she is prescribed a three-day “reflection” period, which causes her distress and affects her relationship with her partner. In *La inocencia*, although abortion is legal, the film problematises the situation of the protagonist, a minor who first resorts to alternative solutions out of fear of telling her parents.
(4) Backstreet abortions performed illegally and resulting in complications, violence or even the death of pregnant women (*O corno, Las buenas compañías*). Abortion is presented as a risky, illegal and stigmatised practice. The protagonists are young women, whose lives unfold in historical contexts in which abortion is illegal, underscoring the political and structural dimension of the criminalisation of abortion.
(5) Unwanted motherhoods, as a result of pregnancies carried to term against the pregnant woman’s will (*Culpa, La maternal*). In *La maternal*, this narrative shows a young, socially and economically vulnerable protagonist. In *Culpa*, the lack of knowledge of the pregnancy until it is at quite an advanced state is linked to the trauma of rape, leading to the protagonist’s social isolation and disconnection from her own body.
(6) Illegal adoptions resulting from unwanted pregnancies in vulnerable women (*La hija, La jefa*). These films depict situations of abuse of power in which young women lacking resources and family support are forced or manipulated into giving their children up for unregulated adoption. These appropriations of the newborn are usually carried out by figures with economic or symbolic power, who take advantage of the precariousness of the pregnant women’s situations.
(7) “Cautionary” narratives, in which unwanted pregnancy and backstreet abortions are linked to death and/or suicide (*Las buenas compañías, O corno*). This type of story takes on a tragic tone, in which death is presented as the inevitable outcome of an unwanted pregnancy in a hostile context.

Apart from these narratives, other relevant results were found, including:

(8) Frustrated motherhoods, due to failed fertilisation processes or medical errors (*Mamífera, La noche de las dos lunas, Madres paralelas*). Notably, some of the films also feature narratives that, through secondary characters, deal with the impossibility of achieving a wanted pregnancy, whether due to infertility or medical negligence. The protagonists are usually middle- or upper-class women, with access to reproductive treatments. In this sense, the films highlight a less frequently addressed aspect of the motherhood narrative: reproductive frustration in contexts of a certain level of privilege.
(9) Lack of “restoration of order” narratives. We refer to a conventional narrative structure in which an initial disruption – such as an unplanned pregnancy – is ultimately resolved in a way that reaffirms social norms and restores equilibrium, often without requiring the protagonist to make a transformative decision. A common example of this device is the “pregnancy scare”, understood here as a situation in which a character temporarily believes she is pregnant, prompting a reflection on possible reproductive choices, but ultimately discovers that she is not. Unlike in the films from earlier decades,^54^ no story was found that used a pregnancy scare as a narrative device to discuss the possibility of terminating a pregnancy without the protagonist ultimately having to make a decision.

### Recurring content and discourses

Most characters considering abortion have marginalising traits, often linked to social, gender, class, or racial factors (*La maternal, Paula*). Some are symbolically represented, such as isolated characters (*Culpa, O corno*) or those who don’t meet societal expectations (*La noche de las dos lunas, Mamífera*). These traits make them vulnerable to judgment or the authority of patriarchal institutions, such as family, state laws, medical systems, employers, or the Church. Many unwanted pregnancies stem from abuse of power, including rape (*Culpa, Madres Paralelas, La señora Brackets, La niñera, El nieto bastardo, Emma Suárez*) or abusive employer-employee relationships (*Paula*). Notably, rapes are committed by familiar figures, suggesting unwanted pregnancy is more common than extraordinary (*Las buenas compañías* even hints at familial rape). State or social services can also perpetrate abuse, such as illegal adoptions within the character’s close circle (*La hija*). In these contexts, protective environments are lacking. Whether through family ties (*Mamífera*, *Els dies que vindran*, *Madres paralelas*, *La inocencia*) or forms of solidarity between women (e.g. *O corno*, *La maternal*, *La hija*), support networks are present but often limited or fragile. In many cases, these relationships do not translate into sustained support throughout the reproductive decision-making process, and experiences of unplanned pregnancy and abortion remain marked by isolation. These forms of what may be described as “situational sisterhood” – temporary alliances shaped by specific circumstances – offer moments of care or assistance, but are frequently constrained by social, legal, or emotional factors. Assistance often comes from marginalised figures, such as healers, midwives, or older women who operate outside legal frameworks and are frequently associated with risk or death (e.g. *La noche de las dos lunas*, *Paula*, *Las buenas compañías*, *La inocencia*, *O corno*). As a result, even when support is present, reproductive experiences are often lived in conditions of partial or structural solitude.

### Representations and significant filmmaking elements

#### Non-representation of the abortion

Representational ellipsis and the use of off-camera space are strategies common to all the films featuring an abortion, whether they offer a dramatic view of pregnancy termination or approach it from more everyday, contemporary and progressive perspectives. This happens significantly in both cases of backstreet abortions resulting in the suffering and even death of the pregnant woman (*O corno, Las buenas compañías*) and those that take place within the safety of the legal framework and in a way that is not necessarily violent for pregnant women (*Mamífera, La inocencia*). In most cases, the abortion is suggested through the use of metaphors and metonymies. Whereas metaphors evoke abortion through symbolic imagery, metonymies suggest it indirectly by showing associated objects, actions, or events rather than the procedure itself. Examples of such metaphors include the dreams of the pregnant woman in *Mamífera*, as an abstraction of her decision to terminate the pregnancy, or the image of a plane dusting crops in Paula, a metaphor for the protagonist’s attempt to get an illegal abortion.

As for metonymies, abortion-inducing objects and instruments make recurring appearances. First, there is the home-brewed abortifacient, a mysterious concoction usually made from plants and administered by an equally mysterious female figure with a matriarchal air (*La noche de las dos lunas, Paula, O corno*). Both elements take centre stage in *O corno*, in which the woman performing the abortion is the protagonist and the abortive plant, the rye ergot from which the film takes its title.

Another meaningful example of metonymy is the coat hanger as a tool for performing backstreet abortions, often self-inflicted and directly related to violence against or even the death of pregnant women (*O corno, Las buenas compañías*). In these cases, the metonymy is not filmed either, but rather exists only at the level of the verbal account or through the physical scars left on the bodies of the women who have been subjected to it (*O corno*).

#### Blood as a visual motif

The physical symptoms of abortion, particularly pain and bleeding, are recurring metonymic devices in the films analysed (e.g. *O corno*). Rather than depicting the abortion procedure itself, these films frequently rely on bleeding to evoke its physical and emotional consequences. Blood thus becomes a significant visual motif, functioning as a recurring element of the mise en scène^62^ and acquiring narrative and symbolic meaning beyond the immediate plot.^65^ Its repeated presence contributes to constructing abortion as a bodily experience, even when the procedure itself remains outside the frame.

Blood is ambiguous due to its narrative polysemy, as it appears in various reproductive processes: menarche, menstruation, miscarriage, and abortion. In *Paula*, the protagonist initially believes bleeding means a miscarriage, only to discover it was implantation bleeding. In *La noche de las dos lunas*, blood on a performer’s thigh suggests a miscarriage. In *La maternal*, the teenage protagonist doesn’t connect her missed period to pregnancy until it's too late. In these cases, the lack of knowledge or resources to understand their physical changes leads to unwanted pregnancies. Taken together, these representations suggest that the films engage with broader issues of sexual and reproductive health literacy.

#### Spaces and settings: isolation and secrecy

An important filmmaking element is the staging and locations of unwanted pregnancy and abortion, often tied to loneliness, isolation, secrecy, or escape. Whether viewed as a crime or taboo, abortion is associated with stigma,^66^ something experienced in solitude and avoidance. This theme of isolation spans from women travelling for a safe abortion (*La inocencia, Las buenas compañías*) to young women cut off from their families to protect honour (*Madres paralelas, Las buenas compañías, La señora Brackets, La niñera, El nieto bastardo, Emma Suárez*), illegal abortion practitioners fleeing the law (*O corno*), or victims of power abuses facing extreme survival situations like kidnapping or exile (*Culpa, La hija, La jefa*). Symbolic exile spaces, such as nature or remote rural areas, emphasise isolation, while domestic settings become prisons, representing forced (*La hija*) or voluntary (*Mamífera*) confinement.

### Narrative positioning and underlying discourses

Beyond narrative outcomes, it is important to examine how abortion is framed at a discursive level. Similar events – such as unsafe abortions or the death of a pregnant woman – can convey different meanings depending on tone, character alignment, and narrative context. These representations do not necessarily operate as direct stigmatisation, as they often reflect historical contexts in which abortion was illegal and unsafe. However, the recurrent association of abortion with risk, danger, or death contributes to the persistence of negative imaginaries, particularly in the absence of alternative representations that normalise it.

In some films (e.g. *O corno*, *Las buenas compañías*), unsafe abortion is framed as a consequence of restrictive legal and social conditions, emphasising vulnerability and lack of access to care. In these cases, suffering is presented less as moral punishment than as an effect of structural constraints. In others (e.g. *La noche de las dos lunas*), reproductive decision-making is embedded in more ambiguous or cautionary narratives, where outcomes are shaped by uncertainty, secrecy, or unintended consequences, without an explicit critique of structural conditions. Overall, abortion is rarely depicted as a normalised experience, but rather as embedded in conflict, danger, or exceptionality. Distinguishing these narrative positions allows for a more nuanced understanding of how abortion is culturally constructed and how these representations intersect with debates on reproductive rights and access to care.

These findings are relevant to sexual and reproductive health and rights debates as they reveal how cinema participates in the cultural regulation of reproductive decision-making. Across the films analysed, abortion is rarely framed as a legitimate and ordinary outcome; instead, it is displaced into secrecy, risk, delay, or exceptional conflict. While films do not determine individual behaviour, they contribute to the symbolic conditions under which abortion becomes thinkable, speakable, and socially acceptable – or remains stigmatised and marginal.

The analysis further shows that reproductive agency is unequally distributed and deeply structured by socio-economic conditions. Characters with greater resources are more likely to access autonomous reproductive choices, whereas those in vulnerable positions are disproportionately associated with constrained motherhood, failed abortion attempts, or coercive outcomes. In this sense, these representations echo a central concern in global health and reproductive justice: reproductive choice is not exercised on equal terms, but is shaped by structural inequalities that condition access to care and the very possibility of deciding.

## Conclusions

This research draws two main conclusions about unplanned pregnancy narratives in contemporary Spanish cinema. First, while the number of films addressing unplanned pregnancies has increased, abortion remains a minority topic. Second, economic and cultural factors significantly shape women's sexual and reproductive health experiences.

The analysis shows that, despite the rise in films depicting unplanned pregnancies, the narratives often reproduce normative understandings of reproduction, privileging motherhood as the expected outcome of pregnancy while limiting the representation of abortion as a valid reproductive choice. Most films explore two perspectives: wanted motherhood or the impossibility of accessing abortion. Legal abortion is still a minority representation, seen in only two of the analysed films. One possible explanation for this limited presence lies in narrative conventions: legal and accessible abortion, particularly when experienced without major conflict, may be perceived as less dramatically compelling and therefore less frequently developed as a central plotline. However, this tendency also reflects broader cultural and ideological frameworks that have historically problematised abortion, favouring narratives centred on conflict, moral tension, or eventual motherhood over those that normalise abortion as a routine and legitimate reproductive outcome. From a rights-based perspective, these findings highlight that the formal recognition of abortion rights does not guarantee their effective exercise, which remains conditioned by stigma, inequality, and access to resources.

The socio-demographic diversity of the protagonists is limited, with most being heterosexual, white women. While there is some generational and relationship status diversity, the employment profiles often reflect socio-economic vulnerability. Only two women have stable jobs, with the rest either unemployed or precariously employed, reflecting the traditional association between unplanned pregnancies and socio-economic disadvantage.

The results suggest an increase in women actively making decisions about their reproductive processes. However, this ability is heavily influenced by social, economic, and cultural factors, as illustrated in films like *Paula* and *La maternal*. For instance, a woman's decision to terminate a pregnancy is more accessible for those with cultural privilege and resources. This problematises the use of the term “unwanted pregnancy,” as it tends to individualise the experience and obscure the structural conditions that shape it.

Furthermore, films depicting voluntary pregnancy termination are rare. When abortion is shown, it is often represented symbolically or through omission, with the physical process rarely depicted directly. Silence plays a key role in the portrayal of abortion, highlighting its confinement to the private sphere. The only figures who break this silence are medical professionals and networks of women, which serve as spaces of trust.

Lastly, some narratives still associate abortion with death, reflecting the moralising discourses of the past. This lingering connection points to the ongoing social stigma surrounding abortion, as suggested by Goffman.^66^ Overall, the films analysed demonstrate that unplanned pregnancy is not an individual choice but an experience shaped by structural inequalities, with the ability to decide profoundly influenced by economic, cultural, and symbolic capital.

These findings highlight the importance of addressing the cultural dimensions of sexual and reproductive health and rights. While the period analysed (2014–2024) coincides with significant legal and political developments – including the consolidation and recent reform of abortion rights in Spain^17,56^ and ongoing global efforts to expand access to safe abortion^5^ – cultural representations do not necessarily evolve at the same pace. Contemporary Spanish cinema reveals a persistent tension between these legal advances and the endurance of restrictive imaginaries, in which abortion remains marginal and unwanted motherhood retains greater narrative centrality. In this context, cinema does not simply reflect social change but contributes to shaping how reproductive experiences are understood, by framing abortion as thinkable, contested, or marginal.

These findings underscore that reproductive rights do not operate in a symbolic vacuum. Cultural narratives shape how reproductive experiences are imagined, judged, and made publicly intelligible, influencing stigma, legitimacy, and the conditions under which rights can be exercised. The persistence of narratives centred on constraint, risk, or moral conflict points to a gap between legal recognition and cultural normalisation. This gap highlights a central principle of reproductive justice: the right to decide depends not only on the legal recognition of reproductive rights, but also on the social, economic, cultural, and symbolic conditions that make those rights meaningful and exercisable.

Beyond the Spanish context, these findings are also relevant in light of the increasing international circulation of Spanish audiovisual productions through film festivals, global distribution networks, and streaming platforms, which facilitate the transnational diffusion of reproductive imaginaries. In this sense, representations produced within the Spanish context may contribute to broader cultural understandings of abortion, motherhood, and reproductive decision-making across different regions, including Latin America. Future research could build on this work by undertaking comparative analyses across national contexts, in order to examine how reproductive imaginaries are shaped by specific legal, cultural, and socio-political contexts.

## Author contributions

*Conceptualisation: MVB, MC, JS. Methodology: MVB, MC. Formal analysis: MVB, MC, JS. Investigation: MVB, MC, JS. Data curation: MVB, MC, JS. Funding acquisition: MVB, MC. Project administration: MVB. Supervision: MVB, MC. Writing – original draft: MVB. Writing – review & editing: MVB, MC, JS. Validation: MVB, MC, JS.*

## Data Availability

The data supporting the findings of this study are available from the corresponding author upon reasonable request.

## Footnotes

* https://materscreendatabase.udl.cat/.
